# Spatial distribution of B cells and lymphocyte clusters as a predictor of triple-negative breast cancer outcome

**DOI:** 10.1038/s41523-021-00291-z

**Published:** 2021-07-01

**Authors:** Juliana C. Wortman, Ting-Fang He, Shawn Solomon, Robert Z. Zhang, Anthony Rosario, Roger Wang, Travis Y. Tu, Daniel Schmolze, Yuan Yuan, Susan E. Yost, Xuefei Li, Herbert Levine, Gurinder Atwal, Peter P. Lee, Clare C. Yu

**Affiliations:** 1 Department of Physics and Astronomy, University of California, Irvine, Irvine, CA USA; 2 Department of Immuno-Oncology, City of Hope Comprehensive Cancer Center and Beckman Research Institute, Duarte, CA USA; 3 Department of Pathology, City of Hope Comprehensive Cancer Center, Duarte, CA USA; 4 Department of Medical Oncology and Therapeutics Research, City of Hope Comprehensive Cancer Center, Duarte, CA USA; 5 Department of Bioengineering and the Center for Theoretical Biological Physics, Rice University, Houston, TX USA; 6 Department of Bioengineering and Department of Physics, Northeastern University, Boston, MA USA; 7 Cold Spring Harbor Laboratory, Cold Spring Harbor, NY USA

**Keywords:** Breast cancer, Tumour immunology, Cancer microenvironment, Tumour heterogeneity

## Abstract

While tumor infiltration by CD8^+^ T cells is now widely accepted to predict outcomes, the clinical significance of intratumoral B cells is less clear. We hypothesized that spatial distribution rather than density of B cells within tumors may provide prognostic significance. We developed statistical techniques (fractal dimension differences and a box-counting method ‘occupancy’) to analyze the spatial distribution of tumor-infiltrating lymphocytes (TILs) in human triple-negative breast cancer (TNBC). Our results indicate that B cells in good outcome tumors (no recurrence within 5 years) are spatially dispersed, while B cells in poor outcome tumors (recurrence within 3 years) are more confined. While most TILs are located within the stroma, increased numbers of spatially dispersed lymphocytes within cancer cell islands are associated with a good prognosis. B cells and T cells often form lymphocyte clusters (LCs) identified via density-based clustering. LCs consist either of T cells only or heterotypic mixtures of B and T cells. Pure B cell LCs were negligible in number. Compared to tertiary lymphoid structures (TLS), LCs have fewer lymphocytes at lower densities. Both types of LCs are more abundant and more spatially dispersed in good outcomes compared to poor outcome tumors. Heterotypic LCs in good outcome tumors are smaller and more numerous compared to poor outcome. Heterotypic LCs are also closer to cancer islands in a good outcome, with LC size decreasing as they get closer to cancer cell islands. These results illuminate the significance of the spatial distribution of B cells and LCs within tumors.

## Introduction

The evolution and progression of cancer is dependent on the tumor microenvironment (TME), which consists of cancer cells, collagen fibers, blood vessels, lymph vessels, fibroblasts, and various types of immune cells. High densities of tumor-infiltrating lymphocytes (TILs) correlate with favorable clinical outcomes in different types of cancer^1–3^, including triple-negative and HER2-positive breast cancers^4^. While the prognostic value of the density of CD3^+^ and CD8^+^ T cells infiltrating tumors is well known, e.g., recurrence in colorectal carcinoma^5^, the clinical significance of B cell density in tumors is less clear^6,7^. Recent studies indicate that B cells in tumors, especially in conjunction with organized lymphoid aggregates known as tertiary lymphoid structures (TLS), are significant predictors of response to immunotherapy, i.e., immune checkpoint blockade, in melanoma^8,9^, soft tissue sarcomas^10^, and renal cell carcinoma^9^. However, the possible clinical relevance of non-TLS B cells in tumors should not be overlooked as B cells can perform a variety of functions, including antibody production, cytokine secretion, and antigen presentation to helper T cells.

**Fig. 1:**
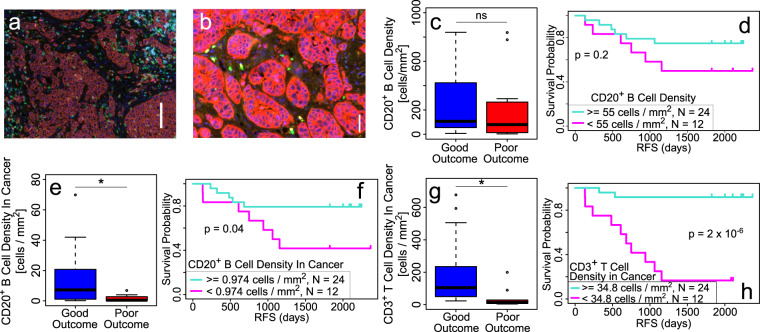
Overall CD20^+^ B cell density is not clinically significant, but the density of TILs within cancer cell islands is significant. **a** Sample image of TNBC tissue illustrating the heterogeneity of the tumor microenvironment. Color code: T cells (CD3^+^; green), B cells (CD20^+^; cyan), cytotoxic T cells (CD8^+^; bright red), regulatory T cells (FoxP3^+^, magenta), cancer cells (Pan-cytokeratin cells; dark brownish red), and nuclei (DAPI; dark blue). Cancer cell islands correspond to clusters of cancer cells (dark brownish red). Scale bar = 100 μm. **b** Example of cancer cell islands interspersed with stroma. Color code: T cells (CD3^+^; green), B cells (CD20^+^; cyan), cancer cells (Pan-cytokeratin cells; red), and nuclei (DAPI; dark blue). Scale bar = 50 μm. **c**, **d** The density of B cells overall in tumor does not differentiate between good vs. poor outcome. **c** Box and whisker plot showing CD20^+^ B cell density in the entire tumor is not significantly different between good (*n* = 24) vs. poor (*n* = 12) outcome. Center line represents the median, box limits represent the upper and lower quartiles, whiskers indicate maximum and minimum values within 1.5 interquartile ranges of the upper and lower quartiles; individual points are outliers. **d** Relapse-free survival (RFS) plot showing that CD20^+^ B cell density in the entire tumor is not a significant factor in TNBC clinical outcome, using a threshold value of 55 cells/mm^2^ as described in “Methods”. **e**–**g** Density of B and T cells within cancer cell islands is clinically significant. **e** Plot showing density of CD20^+^ B cells within cancer cell islands is higher for good (*n* = 24) vs. poor (*n* = 12) outcome (*p* = 0.02). Center line represents the median, box limits represent the upper and lower quartiles, whiskers indicate maximum and minimum values within 1.5 interquartile ranges of the upper and lower quartiles; individual points are outliers. **f** RFS plot showing the clinical significance of density of CD20^+^ B cells within cancer cell islands using a threshold value of 0.974 cells/mm^2^ (see “Methods”). **g** Plot showing density of CD3^+^ T cells within cancer cell islands is higher for good (*n* = 24) vs. poor (*n* = 12) outcome (*p* = 0.01). Center line represents the median, box limits represent the upper and lower quartiles, whiskers indicate maximum and minimum values within 1.5 interquartile ranges of the upper and lower quartiles; individual points are outliers. **h** RFS plot showing the clinical significance of density of CD3^+^ T cells within cancer cell islands using a threshold value of 34.8 cells/mm^2^ (see “Methods”). *P*-values in box plots are determined by unpaired, two-tailed Student *t*-tests. ns: not significant; *p < 0.05; **p < 0.01; ***p < 0.001; ****p < 1 × 10^−4^.

We hypothesized that the spatial patterns of B cells within tumors may be more informative than density alone. Focusing on cell densities averaged over the entire tissue overlooks the possible significance of spatial heterogeneity of TILs^11–13^. Furthermore, spatial heterogeneity could skew the density measured in tissue microarrays (TMA), which only account for a small region within each tumor—such samples may give a poor estimate of the overall cell density, depending on the location of each TMA sample within the tissue. In addition, the TME is spatially heterogeneous with aggregates of cancer cells (cancer cell islands) interspersed with stroma (Fig. 1a, b). In this paper, we utilized density-based clustering algorithm, occupancy, and fractal dimension (FD) approaches to investigate the clinical relevance of the spatial distribution of B and T cells as well as lymphocyte clusters (LCs) in triple-negative breast cancer (TNBC) tumors.

## Results

### Patient cohort and tissue sample preparation

We examined immunohistochemistry (IHC)-based images of primary TNBC tumors (Fig. 1a, b) from 36 patients prior to treatment. Patients with no recurrence within 5 years after surgery were defined as good clinical outcome (*n* = 24) and patients who had recurrence within 3 years after surgery as poor clinical outcome (*n* = 12). All patients received standard of care treatments, which included chemotherapy and also radiotherapy in some. None received treatment prior to tumor resection. (See Table 1 for patient clinicopathological characteristics.) Normal breast tissues from nine patients who underwent breast reduction surgery (age 18–45) served as controls.

**Table 1:** Clinicopathological characteristics of patients with TNBC.

	Good outcome	Poor outcome	Normal tissue
# of Patients	24	12	9
Mean age	55	58	24
Age range	27–76	46–79	18–45
Stage I	5	6	
Stage II	17	6	
Stage III	2	0	
T1	7	6	
T2	16	6	
T3	1	0	
N0	19	10	
N1	3	2	
N2	2	0	
M0	3	0	
M1	0	0	
MX	21	12	
Grade 1	0	0	
Grade 2	3	0	
Grade 3	21	12	
Mastectomy	15	2	
Breast-conserving surgery	9	10	

In the TNM classification, T denotes tumor size, N denotes lymph node invasion, and M denotes the metastasis status.

Formalin-fixed paraffin-embedded (FFPE) tumor tissues were sectioned (i.e., 3–5 μm per slide) and baked onto glass microscope slides. The following cell phenotypes were identified on the same slide: T cells (CD3^+^), B cells (CD20^+^), epithelial/cancer cells (PanCK), or “other” (*n* = 36). Cells were counterstained with DAPI to show the locations of nuclei. In an adjacent slide, a subset of this cohort (27 patients: *n* = 19 good, *n* = 8 poor) was co-stained for CD20^+^ B cells, CD8^+^ cytotoxic T cells, CD4^+^Foxp3^+^ regulatory T cells (Tregs), and CD4^+^Foxp3^−^ helper T cells (Th). In this cohort, we will regard CD8^+^ cytotoxic T cells, CD4^+^Foxp3^+^ regulatory T cells, and CD4^+^Foxp3^−^ helper T cells collectively as CD3^+^ T cells. A pathologist delineated tumor regions that were deemed representative of the entire tumor in terms of cellularity and TIL distribution, and that were free of artifacts such as tissue folding. Areas of necrotic tumor were avoided, as were peri-tumoral lymphoid aggregates not in close proximity to tumor cells. Given these constraints, regions were chosen to maximize tumor area.

The mean and median values for various quantities were calculated for each patient and then the average of these values for a given cohort, e.g., good outcome patients, was reported. Statistical significance was ascertained using the two-sided Student’s *t*-test *p*-value (*p* < 0.05) to reject the null hypothesis, and receiver operating characteristic (ROC) area under the curve (AUC), both standard statistical measures of a binary classifier^14^.

### CD20^+^ B cell density in entire tumors is not clinically significant

Consistent with previous studies^6,7^, mean CD20^+^ B cell density in tumors was not significantly different between good and poor outcome (good (*n* = 24): 2.4 × 10^2^/mm^2^; poor (*n* = 12): 2.2 × 10^2^/mm^2^; *p* = 0.8, ROC AUC = 0.6) (Fig. 1c). Relapse-free survival (RFS) curves using a B cell density threshold of 55 cells/mm^2^ (see Methods) were non-significant for clinical outcome patients (Fig. 1d).

### Densities of CD20^+^ B and CD3^+^ T cells within cancer cell islands are significantly higher in good outcome

While the clinical significance of B cell density within the entire tumor tissue was not significant, this does not preclude the possible clinical significance of local density of B cells within distinct locations, i.e., in cancer islands (CI) or stroma. Indeed, the density of CD20^+^ B cells in cancer cell islands (defined as the ratio of the number of B cells in cancer cell islands to the area of the imaged tissue inside the pathologist’s outline, i.e., the region of interest (ROI)) was significantly higher in good compared to poor outcome (good (*n* = 24): 23 B cells/mm^2^; poor (*n* = 12): 1.9 B cells/mm^2^; *p* = 0.002) (Fig. 1e). Clinical significance is shown in the RFS plot in Fig. 1f. For comparison, we found that the CI density of CD3^+^ T cells was also significantly higher in good outcome (good (*n* = 24): 29 T cells/mm^2^; poor (*n* = 12): 56 T cells/mm^2^; *p* = 0.007) (Fig. 1g). In contrast, the density of CD20^+^ B cells in stroma (defined as the ratio of the number of B cells in the stroma to the area of the ROI), was not clinically significant (good (*n* = 24): 33 stromal B cells/mm^2^; poor (*n* = 12): 27 stromal B cells/mm^2^; *p* = 0.6).

### B cells in stroma are more spatially dispersed in good clinical outcome

Although the density of CD20^+^ B cells in stroma is not clinically significant, we hypothesized that their spatial patterns may be informative. We investigated the spatial distribution of CD20^+^ B cells by developing two statistical techniques: occupancy and FD difference^15,16^. Both techniques are quantitative measures of the spatial dispersion of cells. We use the terms “spread out”, “spread apart”, and “spatial dispersion” synonymously to connote both separations between cells as well as coverage of the tissue or tissue region in question since lymphocytes are not contiguous throughout the entire tissue. (For example, confluent cells in vitro are spread out over an entire region but without any separation between adjacent cells.)

**Fig. 2:**
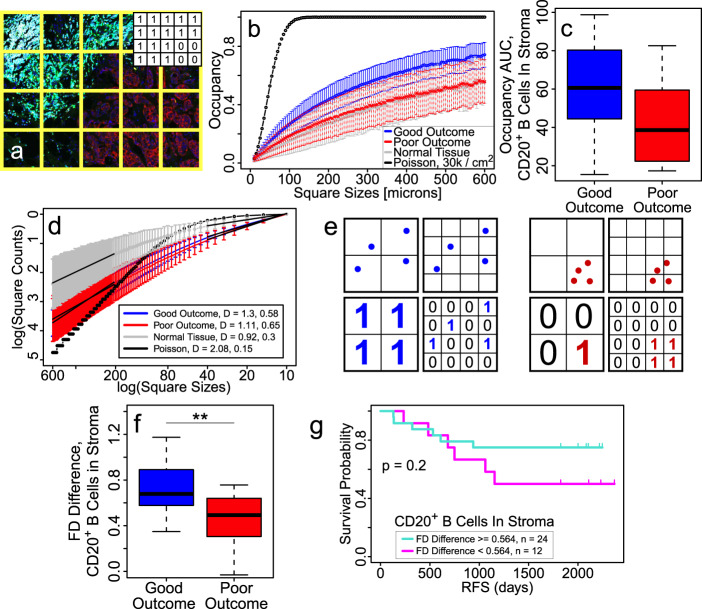
Occupancy, fractal dimensions, and FD differences indicate that stromal B cells are more spread out in good outcome. **a**–**c** Occupancy: **a** Example image showing how occupancy is computed. A grid of squares placed over the image. (Inset) 1’s (0’s) correspond to yes (no) answers to a binary question asked of each square, e.g., “Is there at least one B cell in the square?” Occupancy is fraction of squares with 1’s. **b** Plot of CD20^+^ B cell occupancy in stroma vs. square size for good clinical outcome (blue), poor clinical outcome (red), normal breast tissue (black) and points randomly distributed according to a uniform Poisson process with a density of 3 × 10^2^ points/mm^2^ (cyan). Error bars indicate 95% confidence intervals. Good (blue) outcome curve is consistently higher than poor (red) outcome curve. **c** Plots showing occupancy area under the curve (AUC) for CD20^+^ B cells in stroma is higher for good (*n* = 24) vs. poor (*n* = 12) outcome (*p* = 0.05). Center line represents the median, box limits represent the upper and lower quartiles, whiskers indicate maximum and minimum values within 1.5 interquartile ranges of the upper and lower quartiles; individual points are outliers. **d** Fractal dimension determined from slope of the log-log plot of the number of squares with at least one stromal CD20^+^ B cell vs. the inverse box size (Logarithms are base *e*). The fractal dimension is larger for good clinical outcome vs. poor outcome at large length scales, but this trend is reversed at small length scales. At long length scales (200–600 μm on the left side of the plot), mean fractal dimension *s* (slope) is 1.3 for good outcome (blue), 1.11 for poor outcome (red), 0.92 for normal tissue (gray), and 2.08 for Poisson (black). The *p* value for good vs. poor outcome is 0.08 at long length scales. At short length scales (10–40 μm on the right side of the plot), the mean fractal dimension is 0.58 for good outcome, 0.65 for poor outcome, 0.30 for normal tissue, and 0.15 for Poisson. The *p* value for good vs. poor outcome is 0.5 at short length scales. Black dashed lines show least-squares linear regression fit at long and short length scales. Because different images have different overall areas, fractal dimension is calculated by normalizing the number n(L) of boxes with 1’s with the total number N(L) of boxes with cells. Thus the y-axis values are negative. The error bars correspond to 95% confidence intervals. **e** Example illustrating how FD difference indicates whether cells are spread out or clustered. The left image with four quadrants shows four blue points that are spatially spread out in the upper two quadrants while the right image with four quadrants shows four clustered red points in the upper two quadrants. At long length scales (big boxes in left 2 quadrants), the big squares in the lower left quadrant have more 1 s in the left image than in the lower left quadrant of the right image because the blue points in the upper left quadrant are more spread out compared to the red points in the upper left quadrant of the right image, so the fractal dimension is larger in the left half of left image than in the left half of the right image. At small length scales (small boxes in 2 right quadrants), the upper right quadrants of both images have the same number of points and occupy the same number of squares; hence, the blue and red points have the same fractal dimension at small length scales. So the difference in fractal dimension between large and small length scales will be larger for the spatially dispersed blue points than for the clustered red points. **f** Plot showing that the FD difference for stromal CD20^+^ B cells is significantly higher in good outcome (*p* = 3 × 10^−3^, ROC AUC = 0.77), indicating that B cells are more spatially dispersed in the stroma in good outcome. Center line represents the median, box limits represent the upper and lower quartiles, whiskers indicate maximum and minimum values within 1.5 interquartile ranges of the upper and lower quartiles; individual points are outliers. **g** RFS plot showing the clinical significance of the FD difference for stromal CD20^+^ B cells with a threshold value = 0.564 (see “Methods”). For all FD differences calculated in this paper, the large length scale range is 200–600 μm and the small length scale range is 10–40 μm. Patient cohorts for all plots in this figure are *n* = 24 for good outcome (blue) and *n* = 12 for poor outcome (red). *P*-values in box plots are determined by unpaired, two-tailed Student *t*-tests. ns: not significant; *p < 0.05; **p < 0.01; ***p < 0.001; ****p < 1 × 10^−4^.

Using our occupancy analysis (described in “Methods” and Fig. 2a), Fig. 2b is a plot of the average occupancy for CD20^+^ B cells in stroma versus square size L for 36 patients. Of interest, B cells are not randomly Poisson distributed within tumors (black line). Occupancy of CD20^+^ B cells in normal breast tissue (gray) is compared to that of stromal CD20^+^ B cells within tumors from good outcome patients (blue) and poor outcome patients (red). The difference in occupancy between patients with good and poor clinical outcome reflects, to some extent, the minor difference in B cell density since a higher density corresponds to a higher probability that a square will be assigned a ‘1’ in computing occupancy. The area under the occupancy curve (occupancy AUC) is correlated with outcome (*p* = 0.05, ROC AUC = 0.70) as shown in Fig. 2c. The modestly higher value of occupancy for good outcome indicates that CD20^+^ B cells are more spatially dispersed for good outcome and, in contrast, more spatially confined for poor outcome.

### Fractal dimension (FD)

To introduce FD, the number of tiles, each with area L^2^, required to cover the floor of a room is proportional to 1/L^2^, where the exponent 2 reflects the two-dimensional floor. Similarly, the number n(L) of squares with 1’s is proportional to 1/L^d^, where d is one way to define the FD^17^. Our definition of FD is slightly different (see “Methods” and Fig. 2d) and allows us to characterize a range of length scales with a single FD. (“Length scale” refers to the box size. It can also be roughly thought of as the magnification factor. Small length scales correspond to large magnification factor.) Although a number of papers have used FD to analyze morphologies associated with tumors^18–24^, here the purpose is to quantify the spatial distribution of immune cells. Figure 2d shows a plot of ln(n(L)) versus ln(1/L) for stromal CD20^+^ B cells. At small (10–40 μm) and large (200–600 μm) length scales, the slope of straight lines fitting the data on this log-log plot corresponds to the FD. Large FD, i.e., close to two, corresponds to a more spatially uniform and area-filling spatial distribution of cells. FD is larger for the good clinical outcome than for the poor outcome at large length scales, but this trend is reversed at small length scales. This is key to the FD difference described below which demonstrates that CD20^+^ B cells are more spatially dispersed. As expected, randomly placed points (uniform Poisson process) have a FD of two at length scales large compared to the average separation of points. Further details are given in the “Methods” section.

### FD difference indicates whether cells are spatially dispersed or aggregated^15,16^

The spatial dispersion of cells is reflected in the difference Δ*s* in FD between large and small length scales: Δ*s* = *s*_Large_ − *s*_small_, where *s*_Large_ is the FD at large length scales and *s*_small_ is the FD at small length scales. The small and large length scales should roughly bracket the typical, or median, nearest neighbor distance between cells of the same type, e.g., CD20^+^ B cells. Since the mean nearest neighbor distance between B cells is about 60 μm, we determine *s*_small_ (*s*_Large_) over the range 10–40 (200–600)μm. In all cases examined, Δ*s* > 0. Large Δ*s* indicates that cells are more dispersed, i.e., more spatially spread out and separated because they appear more two-dimensional at large length scales and more zero-dimensional (point-like) at small length scales. (Fig. 2e explains why FD difference is a measure of spatial dispersion.) Δ*s* = 0 indicates that FD does not change with length scale, i.e., the system is self-similar and fractal. Small Δ*s* corresponds to a system that is closer to being fractal (see Fig. 2e).

FD difference Δ*s* for stromal CD20^+^ B cells is plotted in Fig. 2f where we see that Δ*s* is large (Δ*s* = 0.72) for good (*n* = 24) outcome, indicating that stromal B cells are spatially spread out, but small (Δ*s* = 0.46) for poor (*n* = 12) outcome, indicating that stromal B cells are spatially confined. Figure 2f shows that FD difference is clinically significant (*p* = 3 × 10^−3^, ROC AUC = 0.77) (see also the RFS plot in Fig. 2g). For stromal CD20^+^ B cells, Δ*s* is not statistically correlated with stromal B cell density (*p* = 0.4, *r* = 0.16), indicating that spatial distribution of B cells is an independent predictor of clinical outcome.

### Spatial distribution of clustered lymphocytes vs. isolated lymphocytes: Identification of LCs with density-based clustering algorithm (DBSCAN)

**Fig. 3:**
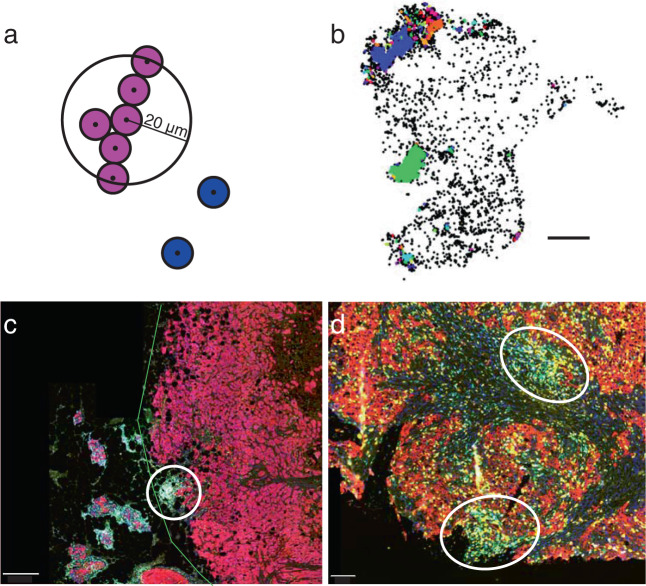
Lymphocyte clusters (LCs) within tumors. **a** Cartoon illustrating density-based clustering algorithm (DBSCAN). LCs contain at least five lymphocytes within a circle with a diameter of 40 μm. **b** Figure illustrating LCs identified in a tumor sample from a poor outcome patient. Each color corresponds to a unique LC. Black points are isolated lymphocytes. **c**, **d** Tumor samples with heterotypic LCs (circled) and isolated lymphocytes from (**c**) poor outcome and (**d**) good outcome. Scale bar = 500 μm in (**c**) and 100 μm in (**d**). Green line is the pathologist’s outline of the tumor region. Color code: Nuclei (DAPI, dark blue), T cells (CD3^+^; green), B cells (CD20^+^; cyan), cytotoxic T cells (CD8^+^; yellow), regulatory T cells (FoxP3^+^, yellow), and cancer cells (Pan-cytokeratin cells; magenta in (**c**) and dark brownish red in (**d**)).

Visual examination of tumors revealed that TILs form clusters, LCs, within the stroma. To quantify LCs and investigate their spatial distribution, we utilized a common density-based clustering algorithm (DBSCAN)^25^. LCs are identified as containing at least five lymphocytes (T and/or B cells) within a circle with a diameter of 40 μm, corresponding to the minimum size containing the centers of five contiguous cells along a line assuming a cell diameter of 10 μm (Fig. 3a). An example with LCs in a scatter plot of lymphocytes is shown in Fig. 3b. LCs consist of T cells only (T LCs) or mixed T and B cells (heterotypic LCs). B cell only LCs constituted <1% of all LCs and were very small (<10 lymphocytes/LC). Figure 3c, d shows heterotypic LCs in sample tissue images from poor and good outcome patients, respectively. Of note, Fig. 3c, d illustrate lymphocytes that are spatially dispersed in good outcome (Fig. 3c) or spatially confined in poor outcome (Fig. 3d). This is consistent with our occupancy and FD difference calculations above. Lymphocytes are either within LCs or isolated, i.e., not part of an LC. Isolated lymphocytes will be considered first.

### Density and spatial dispersion of CD20^+^ B and CD3^+^ T cells infiltrating cancer cell islands are higher in the good outcome than in the poor outcome

**Fig. 4:**
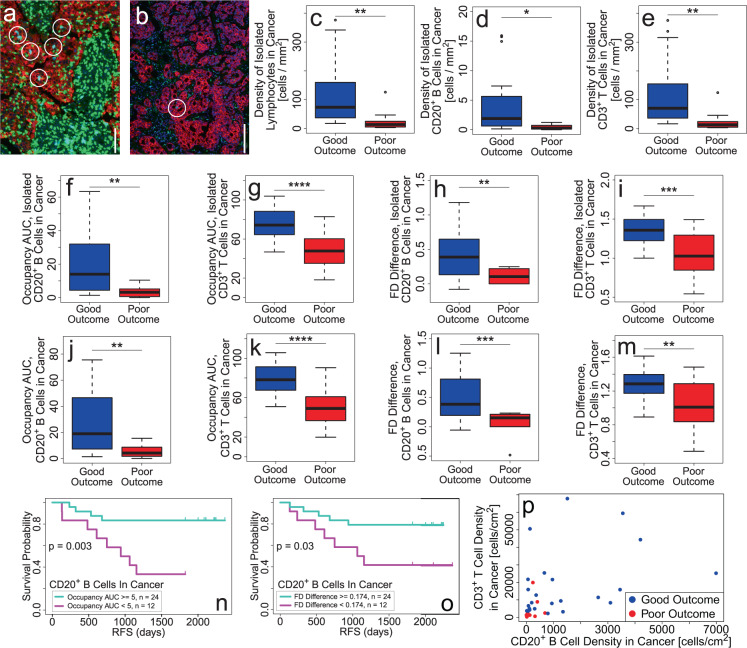
Density and spatial dispersion of B and T cells infiltrating cancer cell islands are higher in good outcome. **a**, **b** Examples of CD20^+^ B cells (circled) within cancer cell islands in (**a**) good (scale bar = 100 μm) and (**b**) poor outcome tumors (scale bar = 200 μm). Color code: T cells (CD3^+^; green), B cells (CD20^+^; cyan), cancer cells (Pan-cytokeratin cells; dark brownish red), and nuclei (DAPI; dark blue). Scale bar = 100 μm (**a**), 200 μm (**b**). **c**–**e** Plots showing density of isolated (**c**) lymphocytes, (**d**) CD20^+^ B cells and (**e**) CD3^+^ T cells infiltrating cancer cell islands is higher for good (*n* = 24) vs. poor (*n* = 12) outcome tumors. **f**–**g** Plots showing occupancy AUC of isolated (**f**) B cells and (**g**) CD3^+^ T cells infiltrating cancer cell islands is higher for good (*n* = 24) vs. poor (*n* = 12) outcome tumors. **h**–**i** Plots showing FD difference of isolated (**h**) B cells and (**i**) CD3^+^ T cells infiltrating cancer cell islands is higher for good (*n* = 24) vs. poor (*n* = 12) outcome tumors. **j**–**o** B and T cells (including both isolated and LC lymphocytes) are more spread out within cancer cell islands in the good outcome: Plots showing occupancy AUC of (**j**) CD20^+^ B cells and (**k**) CD3^+^ T cells within cancer cell islands is higher in good outcome (B cells: *p* = 3 × 10^−3^; T cells: *p* = 6 × 10^−5^). Plots showing FD difference of (**l**) CD20^+^ B and (**m**) CD3^+^ T cells within cancer cell islands is higher for good vs. poor outcome (B cells: *p* = 8 × 10^−4^; T cells: *p* = 2 × 10^−3^). **n** RFS plots showing the clinical significance of occupancy AUC of CD20^+^ B cells within cancer cell islands with a threshold value = 5 corresponding to the fraction of poor outcome patients. **o** RFS plot showing the clinical significance of FD difference of CD20^+^ B cells within cancer cell islands with a threshold value = 0.174 (see “Methods”). **p** Densities of B and T cells (both isolated and in LCs) within cancer islands are significantly correlated (Pearson *r* = 0.45, *p* = 0.006) even if no distinction is made between good and poor outcome. For all box-and-whisker plots, the center line represents the median, box limits represent the upper and lower quartiles, whiskers indicate maximum and minimum values within 1.5 interquartile ranges of the upper and lower quartiles; individual points are outliers. *P*-values in box plots are determined by unpaired, two-tailed Student *t*-tests. ns: not significant; *p < 0.05; **p < 0.01; ***p < 0.001; ****p < 1 × 10^−4^.

The majority of lymphocytes within cancer cell islands are isolated, i.e., not in LCs (good (*n* = 24): 0.64; poor (*n* = 12) 0.76; *p* = 0.04). The density of isolated lymphocytes (*p* = 0.006), as well as isolated CD20^+^ B (*p* = 0.02) and CD3^+^ T cells (*p* = 0.007), infiltrating cancer cell islands is significantly higher in good outcome (*n* = 24) vs. poor outcome (*n* = 12) (Fig. 4a–e). (Density is defined as the ratio of the number of cells in cancer cell islands and the ROI area.) Spatial dispersion of isolated CD20^+^ B and CD3^+^ T cells within cancer cell islands, as measured by occupancy AUC (B cells: *p* = 0.004; CD3^+^ T cells: *p* = 7 × 10^−5^) and FD difference (B cells: *p* = 0.007; CD3^+^ T cells: *p* = 0.0003), is significantly larger in good outcome (Fig. 4f–i).

CD20^+^ B and CD3^+^ T cells within cancer islands, regardless of whether or not they are isolated or clustered, exhibit significantly greater spatial dispersion in good (*n* = 24) vs. poor (*n* = 12) outcome as indicated by occupancy AUC as well as FD difference (Fig. 4j–m). Associated RFS plots in Fig. 4n, o show the clinical significance of CD20^+^ B cell occupancy AUC and FD difference. Densities of CD20^+^ B and CD3^+^ T cells in cancer cell islands, without regard to outcome, are significantly correlated (Fig. 4p).

### Density and spatial dispersion are higher in good outcome vs. poor outcome for isolated CD3^+^ T cells but not isolated CD20^+^ B cells in the stroma

**Fig. 5:**
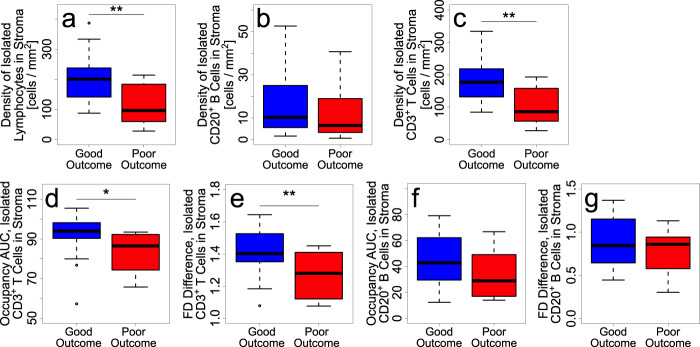
Density and spatial dispersion of isolated lymphocytes and isolated CD3^+^ T cells within stroma are significantly higher for good outcome tumors, but not for isolated stromal CD20^+^ B cells. Density of isolated (**a**) lymphocytes, (**b**) CD20^+^ B cells, and (**c**) CD3^+^ T cells within stroma for good vs. poor outcome tumors. Plots of (**d**) occupancy AUC and (**e**) FD difference of isolated CD3^+^ T cells within stroma showing greater spatial dispersion in good compared to poor outcome. Plots of (**f**) occupancy AUC and (**g**) FD difference showing that spatial dispersion of isolated B cells within stroma is not significantly different between good vs. poor outcome. Patient cohorts for plots in this figure are *n* = 24 for good outcome (blue) and *n* = 12 for poor outcome (red). For all box-and-whisker plots, the center line represents the median, box limits represent the upper and lower quartiles, whiskers indicate maximum and minimum values within 1.5 interquartile ranges of the upper and lower quartiles; individual points are outliers. *P*-values in box plots are determined by unpaired, two-tailed Student *t*-tests. ns: not significant; *p < 0.05; **p < 0.01; ***p < 0.001; ****p < 1 × 10^−4^.

Only a small fraction of lymphocytes in the stroma are isolated (good (*n* = 24): 0.27; poor (*n* = 12) 0.33; *p* = 0.4). While the density of isolated lymphocytes (*p* = 0.004), as well as isolated CD3^+^ T cells (*p* = 0.002), within stroma is significantly higher in good outcome (*n* = 24) vs. poor outcome (*n* = 12) tumors, the density of isolated stromal CD20^+^ B cells is not significant (*p* = 0.5) for outcome (Fig. 5a–c). (Density is defined as the ratio of the number of cells in the stroma and the area of ROI.) In addition, spatial dispersion of isolated CD3^+^ T cells within the stroma, as measured by occupancy AUC (*p* = 0.02) and FD difference (*p* = 0.006), is significantly larger in good vs. poor outcome (Fig. 5d–g). However, spatial dispersion of isolated stromal CD20^+^ B cells is not significant (occupancy AUC: *p* = 0.1; FD difference: *p* = 0.3).

### LCs consist purely of T cells or heterotypic mixtures of B and T cells

LCs, which are almost entirely in the stroma, either consist purely of CD3^+^ T cells or mixtures of CD3^+^ T and CD20^+^ B cells (referred to as “heterotypic LCs”). Less than 1% of LCs are purely B cells and these are very small clusters. Fifty-seven percent of LCs are purely CD3^+^ T cells while 42% are a mixture of T and B cells with no significant difference in these percentages between good and poor outcomes.

### TLS are a clinically insignificant subset of LCs in TNBC tumors

**Fig. 6:**
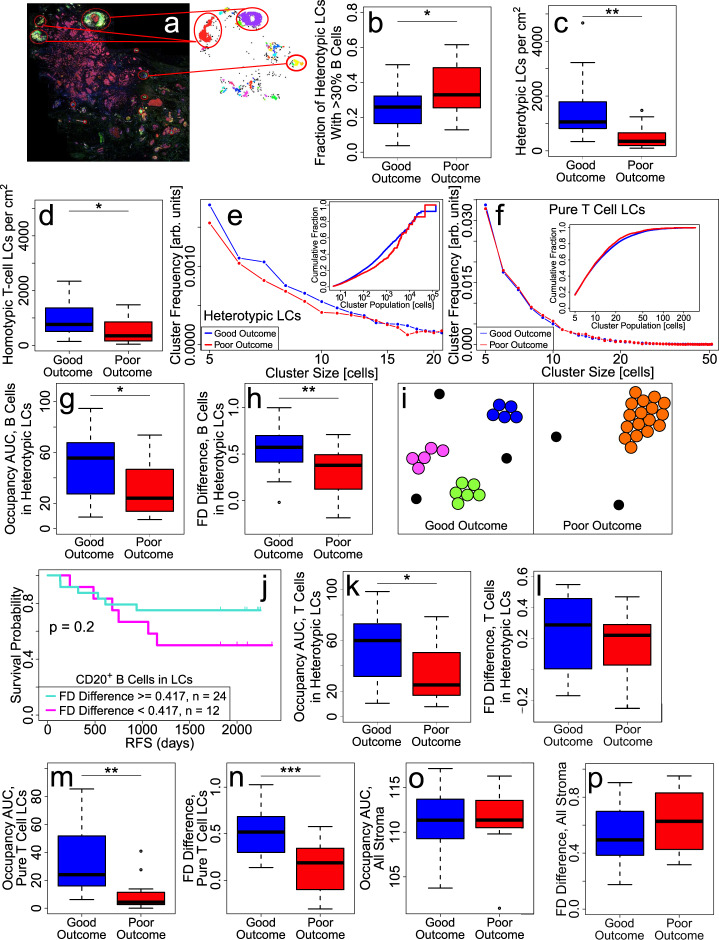
LCs are smaller, more numerous, and more evenly distributed in good outcome. **a** Sample image showing TLS. Color code: Nuclei (DAPI, dark blue), B cells (CD20^+^; yellow), DC-LAMP (magenta), PNAd (green), and cancer cells (Pan-cytokeratin cells; red). **b** Fraction of heterotypic LCs with more than 30% B cells is lower for good (*n* = 24) vs. poor outcome (*n* = 12) tumors. Plots showing (**c**) number of heterotypic LCs per cm^2^ and (**d**) number of pure T cell LCs per cm^2^ are higher for good outcome. **e** Cluster size distribution showing heterotypic LCs are smaller and more numerous in good outcome vs. poor outcome tumors. (Inset) Cumulative fractions of lymphocytes that belong to heterotypic LCs of a given size (population) or smaller show that there are more small clusters in good outcome, and more large clusters in poor outcome, as indicated by crossing of the curves at large cluster sizes. **f** Cluster size distribution of pure T LCs showing no significant difference between good and poor outcome tumors (see “Methods” section for normalization of distributions). (Inset) Cumulative fractions of T cells that belong to pure T cell LCs of a given size (population) or smaller lack distinction between good vs. poor outcome. Plots of (**g**) occupancy AUC of B cells in heterotypic LCs and (**h**) FD difference for B cells in heterotypic LCs, indicating that B cells in heterotypic LCs are more spatially dispersed in good outcome. **i** Cartoon illustrating lymphocytes in LCs that are more spread out (left panel: good outcome) vs. more aggregated (right panel: poor outcome). Each color corresponds to a unique LC. Black points are isolated lymphocytes. **j** RFS plot showing the clinical significance of FD difference of B cells in heterotypic LCs with a threshold value of 0.417 (see “Methods”). **k** Plot showing occupancy AUC of CD3^+^ T cells in heterotypic LCs is higher in good outcome. **l** Plot showing FD difference for CD3^+^ T cells in heterotypic LCs is not significantly different between good and poor outcome. Pure T cell LCs are more evenly distributed in good outcome: (**m**) occupancy AUC and (**n**) FD difference of T cells in pure T cell LCs is higher in good outcome. Spatial distribution of LCs is not attributable to the spatial distribution of stromal cells: (**o**) occupancy AUC and (**p**) FD difference of all stromal cells is not significantly different between good vs. poor outcome. Patient cohorts for all plots in this figure are *n* = 24 for good outcome (blue) and *n* = 12 for poor outcome (red). For all box-and-whisker plots, the center line represents the median, box limits represent the upper and lower quartiles, whiskers indicate maximum and minimum values within 1.5 interquartile ranges of the upper and lower quartiles; individual points are outliers. *P*-values in box plots are determined by unpaired, two-tailed Student *t*-tests. ns: not significant; *p < 0.05; **p < 0.01; ***p < 0.001; ****p < 1 × 10^−4^.

TLS consist of densely packed lymphocytes with a T-cell-rich zone next to a B cell follicle^26^. While TLS are a subset of LCs, most of the LCs identified using our clustering algorithm are morphologically distinct from TLS and not as densely packed. Most LCs have much fewer lymphocytes at a lower density than TLS. The neighbors of lymphocytes in TLS are other lymphocytes while the neighbors of lymphocytes in non-TLS LCs can be a variety of cell types including fibroblasts, cancer cells, and other lymphocytes. Examination of tissue samples immunostained for high endothelial venules (PNAd) and for dendritic cells (DC-LAMP) (good: *n* = 4, poor: *n* = 4) also supports that most LCs are not TLS (data not shown). We defined TLS as containing at least 200 lymphocytes in a circle of radius 70 μm because TLS identified in this way agree with those we can identify visually by immunostaining and with the concept that TLS consist of large groups of densely packed lymphocytes (Fig. 6a).

Recently, TLS together with B cells are predictive of response to immune checkpoint blockade (ICB) in metastatic melanoma tumors^8,9^ and soft tissue sarcomas^10^. In TNBC tumors, we do not observe a significant clinical difference in the fraction of all B cells belonging to TLS (good (*n* = 18): 0.24; poor (*n* = 8): 0.27; *p* = 0.7), the fraction of tissue area occupied by TLS (good (*n* = 18): 0.01; poor (*n* = 8): 0.01; *p* = 0.8), the fraction of all lymphocytes belonging to TLS (good (*n* = 18): 0.11; poor (*n* = 8): 0.14; *p* = 0.5), nor in the fraction of TLS lymphocytes that are B cells (good (*n* = 18): 0.50; poor (*n* = 8): 0.60; *p* = 0.2). Furthermore, most B cells are not in TLS; the majority (about 85%) of B cells are in (heterotypic) LCs. We investigated LC properties, e.g., composition, size, spatial distribution, and location relative to cancer islands, to determine their clinical relevance.

### Most B and CD3^+^ T cells are in heterotypic LCs

The majority of CD20^+^ B cells (good (*n* = 24): 0.85; poor (*n* = 12): 0.84; *p* = 0.7) and CD3^+^ T cells (good (*n* = 24): 0.69; poor (*n* = 12): 0.78; *p* = 0.5) within tumors are found within heterotypic LCs. The fraction of heterotypic LCs with more than 30% B cells is significantly larger in poor outcome (good (*n* = 24): 0.25; poor (*n* = 12): 0.37; *p* = 0.02) (Fig. 6b). This is consistent with the mean relative proportion of B cells in heterotypic LCs being higher in poor outcome (Table 2). The relative proportions of T cells in LCs are shown in Table 2. The lower proportion of B cells complements the higher proportion of CD3^+^ T cells in good outcome since B and CD3^+^ T cell proportions must sum to 1. The higher proportion of T cells is commensurate with the higher density of CD3^+^ T cells in good outcome.

**Table 2:** Relative average proportions of B and T cells in heterotypic LCs, pure T LCs, and TLS.

Cell Type	LC type	Good Outcome (*n* = 19)	Poor Outcome (*n* = 8)	*p* value
CD20^+^ B cells	Heterotypic	0.27	0.38	0.04
Th cells	Heterotypic	0.42	0.38	0.3
CD8^+^ T cells	Heterotypic	0.26	0.19	0.07
Tregs	Heterotypic	0.055	0.050	0.7
Th cells	Pure T	0.53	0.54	0.8
CD8^+^ T cells	Pure T	0.35	0.31	0.3
Tregs	Pure T	0.12	0.15	0.4
CD20^+^ B cells	TLS	0.51	0.62	0.3
Th cells	TLS	0.30	0.21	0.2
CD8^+^ T cells	TLS	0.15	0.15	1.0
Tregs	TLS	0.034	0.023	0.6

In a good outcome, the proportion of T cells is higher while the proportion of B cells is lower. Only lymphocytes are used in computing relative proportions.

### Heterotypic and pure T cell LCs are more abundant in good outcome patients compared to poor outcome patients

Good outcome patients had a significantly higher number of heterotypic LCs per unit area compared to poor outcome (good (*n* = 24): 960/cm^2^; poor (*n* = 12): 520/cm^2^; *p* = 0.04) (Fig. 6c). Similar results hold for pure T cell LCs (good (*n* = 24): 1420/cm^2^; poor (*n* = 12): 517/cm^2^; *p* = 0.005) (Fig. 6d).

### Good outcome has smaller, more numerous heterotypic LCs compared to poor outcome

Distribution of cluster sizes, i.e., number of lymphocytes per cluster, of heterotypic LCs indicates that good outcome tumors have a greater number of smaller clusters than poor outcome tumors (Fig. 6e). Cumulative fraction of lymphocytes that belong to heterotypic LCs of a given size (population) or smaller shows that there are more small clusters in good outcome, and more large clusters in poor outcome, as indicated by crossing of the curves at large cluster sizes (inset of Fig. 6e). Corresponding plot for pure T cell LCs lacks the same distinction between good vs. poor outcome (Fig. 6f). Because the distribution of cluster sizes is skewed, mean and median cluster sizes were not clinically significant for heterotypic LCs (mean: *p* = 0.8, median: *p* = 0.8), though for pure T cell LCs, median size was significant (mean: *p* = 0.6; median: *p* = 0.01). Mann–Whitney test shows that distributions of heterotypic and pure T cell LCs are significantly different (*p* < 2 × 10^−16^). However, pure T cell LCs are typically much smaller than heterotypic LCs.

### B cells in heterotypic LCs are more spread out in good outcome

Spatial dispersion of B (or T) cells in LCs was ascertained using occupancy AUC and FD difference, which were calculated by assigning a ‘1’ to squares that had at least one B (or T) cell that was in a heterotypic LC. Both quantities are significant for B cells (occupancy AUC: *p* = 0.02; FD difference: p = 0.008), indicating that B cells in LCs are more spatially dispersed in good outcome and more spatially confined in poor outcome, similar to our results above for B cells (Fig. 6g, h).

It is not a contradiction that B cells in clusters are more spread out because occupancy and FD difference survey B cells in heterotypic LCs throughout the tissue. Larger spatial dispersion of B cells in LCs in good outcome implies that heterotypic LCs are more spread out and spatially separated compared to poor outcome, where heterotypic LCs are more spatially confined (illustrated in Fig. 6i). In good outcome tumors, heterotypic LCs are smaller and more abundant compared to poor outcome tumors, which have fewer heterotypic LCs with more lymphocytes sequestered per cluster.

The clinical significance of FD difference of B cells in heterotypic LCs is illustrated by the RFS plot in Fig. 6j. For CD3^+^ T cells in heterotypic LCs, occupancy AUC is significant (*p* = 0.01) but FD difference is not significant (*p* = 0.3) (Fig. 6k, l). FD difference of B cells and T cells that are in the same heterotypic LCs differ in significance because higher T cell density results in more squares with 1’s and hence, a higher FD (FD ~ 1) at small length scales (10–40 μm). As a result, the difference in FD between large (200–600 μm) length scales (good (*n* = 24) FD = 1.3; poor (*n* = 12) FD = 1.1; *p* = 0.04) and small length scales will be less for T cells than for B cells in heterotypic LCs. Lower B cell density produces a lower FD for small square sizes in the range of 10–40 μm (good (*n* = 24) FD = 0.67; poor (*n* = 12) FD = 0.78; *p* = 0.2), resulting in a larger difference in FD between large length scales (good (*n* = 24) FD = 1.1; poor (*n* = 12) FD = 0.90; *p* = 0.1) and small length scales.

### T cells in pure T cell LCs are more spatially dispersed in good outcome

A small percentage (about 13%) of CD3^+^ T cells are in pure T cell LCs. There is no significant difference in the fraction of T cells in pure T cell LCs between good vs. poor outcome tumors (*p* = 0.6). However, occupancy AUC and FD differences of pure T cell LCs was significantly higher in good outcome (occupancy AUC: *p* = 3 × 10^−4^; FD difference: *p* = 3 × 10^−4^), indicating that these T cells are more spatially dispersed in good outcome (Fig. 6m, n). Of note, the *p*-values for occupancy AUC and FD differences in pure T cell LCs are about two orders of magnitude more significant than for heterotypic LCs, indicating that spatial distribution of pure T LCs has greater clinical significance. This difference may be related to the density of T cells being a better prognostic indicator than the density of B cells, since the cell density influences occupancy and FD.

### Spatial distribution of LCs is not attributable to spatial distribution of stromal cells

Since a large fraction of lymphocytes in LCs are located within the stroma (good (*n* = 24) 0.80; poor (*n* = 12): 0.91; *p* = 0.05), we investigated whether the spatial distribution of LCs was dictated by exclusion from cancer cell islands. So we computed the occupancy and FD difference of stromal cells which, by definition, are not within cancer cell islands. Occupancy AUC of stromal cells is not significantly different between good vs. poor outcome tumors (*p* = 0.9) (Fig. 6o). Similarly, FD difference is not significantly different for poor outcome vs. good outcome tumors (*p* = 0.3) (Fig. 6p), indicating that stromal cells are similarly distributed in good and poor outcomes. This also indicates that the spatial distribution of lymphocytes that are within LCs, as well as the spatial distribution of B cells overall, is not due to the spatial structure of the stroma in which they reside.

### Heterotypic and pure T cell LCs are closer to cancer cell islands in good outcome tumors

**Fig. 7:**
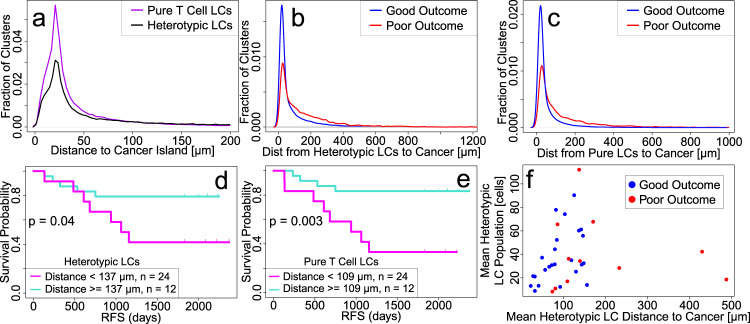
LCs are closer to cancer cell islands in good outcome. **a** Distribution of distances from LCs to cancer islands shows pure T LCs tend to be closer to cancer islands than heterotypic LCs. Distribution of mean distances between an LC and its nearest cancer cell island shows that (**b**) heterotypic and (**c**) pure T cell LCs are closer on average to cancer cell islands in good outcome. RFS plots showing the clinical significance of mean distance between (**d**) heterotypic or (**e**) pure T cell LCs and the nearest cancer cell island. (See “Methods” for how RFS thresholds were chosen.) **f** Smaller heterotypic LCs tend to be closer to cancer cell islands in the good outcome as shown in the scatter plot of the average size (number of cells) of LCs vs. the shortest distance between a heterotypic LC and the nearest cancer cell island. Each point represents a patient. Blue is for good outcome and red is for poor outcome. Pearson’s *r* = 0.42, *p* = 0.04 for good outcome indicates significant correlation while *r* = 0.09, *p* = 0.6 for poor outcome. Patient cohorts for all panels in this figure are *n* = 24 for good outcome (blue) and *n* = 12 for poor outcome (red). Computation of mean distance used in plots: The mean distance is found for each patient and then the average over all patients is calculated to produce the mean distances.

Pure T cell LCs tend to be closer to cancer cell islands than heterotypic LCs, regardless of outcome (Fig. 7a). Distance between each LC and its nearest cancer cell island is defined as the shortest distance between a lymphocyte within the LC and a cell within the nearest cancer island. The mean of such distances is significantly shorter in good outcome compared to poor outcome tumors for both heterotypic and pure T cell LCs (heterotypic LCs: good (*n* = 24): 91 μm; poor (*n* = 12): 200 μm; *p* = 0.002; pure T cell LCs: good (*n* = 24): 73 μm; poor (*n* = 12): 180 μm; *p* = 8 × 10^−5^) (Fig. 7b, c). The clinical significance of the mean distance between LCs and cancer islands is shown in RFS plots in Fig. 7d, e. Furthermore, for good outcome, the size of heterotypic LCs is correlated with their distance from cancer cell islands: smaller LCs have a greater probability of being closer to cancer cell islands (n = 24, Pearson’s *r* = 0.42, *p* = 0.04) (Fig. 7f). This correlation is absent in poor outcomes and for pure T LCs.

## Discussion

Recent studies highlight the clinical significance of B cells in certain human tumors, specifically melanoma and sarcoma^8–10^. However, their role in breast tumors remained unclear. Our results indicate that while the overall density of B cells in TNBC tumors is not clinically significant, B and T cell density within cancer cell islands is significantly higher in good outcome tumors. One speculative reason for TIL infiltration into cancer cell islands is that the cancer islands arise from tumor self-seeding by circulating cancer cells that bring associated lymphocytes with them^27^, (Norton, L., 2019, Private communication). Another possibility is that cytokines and chemokines specifically recruit lymphocytes to cancer islands. In addition, the higher density of B cells infiltrating cancer cell islands in good outcome may be due to a greater diversity of B cell receptors and recognition of cognate tumor antigens. Helmink et al.^9^ found an increased diversity of B cell receptors in responders to immunotherapy compared to non-responders.

Our observation that the density of B and T cells within cancer cell islands is associated with clinical outcome is consistent with previous TNBC studies of neoadjuvant therapy that found densities of both stromal and intraepithelial TILs predictive of pathological response^28,29^. Interestingly, for the luminal subtype of breast cancer, only intraepithelial TILs, not stromal TILs, were indicative of pathological response^29^. However, the established guidelines for evaluating TILs in breast cancer discourage using intraepithelial TILs for prognostication because, compared to stromal TILs, their paucity makes them rarer and harder to detect^30^.

Beyond cell density, B and T cells are more spatially dispersed, within both cancer cell islands and stroma, in good outcome. Consistent with other studies, the majority of TILs are found in stroma. Importantly, most TILs form LCs that are either heterotypic (mixed T and B cells) or pure T cells. LCs comprised of only CD20 + B cells were rare. Both types of LCs are more abundant and more spatially dispersed in good outcome vs. poor outcome. In good outcome tumors, heterotypic LCs are smaller and more numerous compared to poor outcome. These attributes reflect the spatial distribution of LC constituent B and T cells and cannot be attributed to differences in the spatial distribution of cells constituting the stroma where most lymphocytes are found. Heterotypic LCs are closer to cancer islands in good outcome, with LC size (population) decreasing as they get closer to cancer islands. Although segregation of B and T cells is similar in heterotypic LCs and TLS, the number and density of lymphocytes in the majority of heterotypic LCs are too small for these heterotypic LCs to qualify as TLS. Given that TLS with high lymphocyte density are linked to good prognosis, it is somewhat ironic that good outcome is associated with isolated B cells within cancer cell islands and with spatially dispersed B cells, both of which connote low local concentrations of B cells and are the antithesis of high B cell density germinal centers found in mature TLS.

The biological role of B cells within tumors remains unclear. CD20^+^ B cells are not antibody-producing plasma cells since plasma cells do not express CD20. It is possible that CD20^+^ B cells act as antigen-presenting cells (APCs) within tumors. Spatial dispersion of CD20^+^ B cells in good outcome is consistent with their ability to forage over a large region, including in cancer islands, to gather antigens that can be presented to Th cells in heterotypic LCs and TLS. Th cells comprise a substantial fraction of the lymphocytes in TLS and LCs. If CD20^+^ B cells function as APCs, this would be consistent with the paucity of LCs consisting purely of B cells. APC function may also explain why the density of B cells is not correlated with clinical outcome: a single B cell can activate many Th cells if it can spatially disperse.

The source of clinically significant differences between good and poor outcome tumors in the spatial distribution of CD20^+^ B and CD3^+^ T cells is not clear. This does not appear to be due to differences in the physical topography of the stroma as we did not find clinically significant differences in the spatial distribution of stroma. Furthermore, immunohistochemical staining of collagen I did not show differences in the spatial distribution of collagen I between good and poor outcomes (data not shown). Spatially aggregated lymphocytes in poor outcome may be associated with immunoediting, resulting in immune-evasive cancer cell clones^31^. Less spatial dispersion and larger LCs suggest a lack of motility, e.g., in overcoming physical barriers in the stroma such as collagen fibers, due to a lack of chemokine signaling and cognate antigens, or a lack of metabolites due to poorly organized leaky vasculature that produces hypoxic regions, or metabolic insufficiency from dysfunctional depolarized mitochondria that have been associated with exhausted CD8^+^ TILs^32^. It has been suggested that cells are more motile if they are elongated rather than compact and round^33–35^. Future studies should look for correlations between lymphocyte clustering and cell shape, e.g., the aspect ratio or the ratio of the cell perimeter to the square root of the area of a cell^34,35^. Another finding from this study is that the spatial distribution of TILs is fractal. We speculate that this fractal pattern may arise from branching trajectories that B and T cells follow as they avoid obstacles and follow along blood vessels and collagen fibers^36,37^.

Our techniques minimize the noise arising from sparseness of discrete points that correspond to cell locations because they divide the tissue area into squares and ask binary questions that do not depend on the exact location and number of cells in a square. Thus, they effectively average over the larger region of a square. Similarly, density-based clustering, used to identify LCs, depends on the local density of several cells and is basically a form of averaging.

Previous efforts to quantify the spatial heterogeneity of the TME include measuring the spatial co-localization of tumor and immune cells^38,39^, locating immune cell clusters or “hotspots”^40,41^, determining the amount of infiltration of lymphocytes into a tumor^42^, and using Shannon entropy to quantify the cellular diversity^43^. In addition, FDs^44^ have been used to characterize the irregular morphology of tumors^18–20^ and tumor-related structures^21–24^, but not the spatial distribution of immune cells. Spatial heterogeneity connotes local variations in density; this implies a certain size (or length scale) of a region in which density is calculated. All these approaches produce measures of the spatial distribution at a single length scale. The statistical techniques that we have developed move beyond this limitation to quantify how the spatial distribution varies with length scale and then use this to determine whether the cells are clustered or spatially dispersed. On a more general level, these approaches are flexible and applicable to a broad spectrum of problems that go beyond the spatial distribution of cells or discrete entities. More complex binary questions can be employed to explore the proximity of cells and structures indicative of possible function, e.g., regarding co-localization of at least one T cell and a dendritic cell (suggesting antigen presentation by the dendritic cell) or proximity of at least one B cell and one T cell near a blood vessel (suggesting lymphocyte extravasation).

While our results are suggestive, they should be interpreted with caution due to some limitations. The first is the small patient sample size; follow-up studies should involve a much larger cohort of patients. Second, the TNBC patients were somewhat diverse in their disease characteristics and were not uniformly treated after tumor resection, though none had treatment before surgery.

Nevertheless, our results indicate the importance of the spatial distribution of B cells within human TNBC tumors with regard to clinical outcomes. These findings raise new questions about the biology and clinical impact of TILs. Of particular interest is the discordant significance of B cell density vs. spatial distribution on the outcome of TNBC. The role of B cells within tumors is understudied and remains unclear, partially due to prior reports that their density within tumors is of no significance. Our finding of the strong correlation of B cell spatial distribution with clinical outcome in TNBC revives their significance in cancer, and opens up new questions on their functional role in preventing recurrence.

## Methods

### Breast cancer tissue sample preparation and analysis

#### Tissue preparation, multi-color antibody staining, and image analysis

Specimens were identified through an IRB-approved protocol via the City of Hope (COH) Biospecimen Repository which is funded in part by the National Cancer Institute. The IRB granted a waiver of informed consent from all human participants because de-identified tissue samples were obtained from the CoH Biospecimen Repository. Samples from patients diagnosed with TNBC and treated at COH from 1 January 1994 to 4 March 2015 were retrieved. Eligible patients had the following features: stage I-III breast cancer; at least one tumor biospecimen was available from the initial surgical resection or biopsy; clinical outcome data were available for identification of relapse-free survival; no prior treatment at the time of surgical biopsy. Archived FFPE tumor tissues were sectioned (i.e., 3–5 μm per slide) and on the same slide, multi-color immunostaining was performed including anti-pan cytokeratin (1:2850, Cat. # M351501-2, AE1/AE3, Dako), anti-CD8 (1:250, Cat. # CRM 311C, SP16, Biocare), anti-CD3 (1:1000, Cat. # A045201-2, Polyclonal, Dako), anti-FOXP3 (Ready-To-Use, Cat. # API 3164 AA, 236A/E7, Biocare) and anti-CD20 (1:450, Cat. # M075501-2, L26, Dako). Samples were further counterstained with DAPI to visualize the nuclei of all cells. Prior to imaging, the tissue sections were coverslipped with ProLong^®^ Gold Antifade mounting media (Cat. # P36930, Life Technologies). All the images were acquired using the Vectra 3.0 Automated Quantitative Pathology Imaging System (Akoya Biosciences) and commercially available software packages (inForm, Akoya Biosciences) were used to identify each cell, define its type (cancer or specific immune), and assign it Cartesian coordinates. Using an automated tissue segmenter algorithm built in inForm®, we further divided the images into areas of cancer islands and stroma based on anti-pan cytokeratin antibody staining.

#### Spatial distribution analysis of TILs

Using the data spreadsheets with every cell phenotyped and given a set of Cartesian coordinates, we tiled each image with a grid of identical squares. We omitted squares that had no cells, since they lay outside the tissue. However, we included squares that were within the tissue but contained no cells of interest. The length L of one side of a square varies from 10 to 600 μm. A square was assigned 1 or 0 according to the answer to a binary question. For comparison we calculated the occupancy FD of a uniform random (Poisson) distribution of points. We processed the data using in-house custom-developed software (RStudio) and the R packages “spatstat” for point patterns, “dbscan” for cluster analysis, and “EBImage” for raster images^45,46^.

### Statistical methods

#### Occupancy^15,16^

An image is overlaid with a grid of squares (Fig. 2a); each square has an area of *L*^2^. A ‘0’ or ‘1’ is assigned to each square according to the answer to a binary (yes-no) question, e.g., “Is there at least one B cell in the square?” (Fig. 2a inset). The occupancy is the fraction of squares with 1’s, i.e., it is an estimate of the probability that a square will have a 1. To characterize the spatial distribution at different length scales, the size of the squares in the grid is varied to produce the occupancy as a function of L, the length of one side of a square. The occupancy depends to some extent on the average cell density for binary questions such as “Is there at least one CD20^+^ B cell in the square?” because a higher average cell density corresponds to a higher probability that a square is assigned a ‘1’.

#### Fractal dimension (FD)^15,16^

FDs can be used to characterize the number of grid squares with 1’s and hence, the spatial distribution of cells. While there are a number of different ways to define the FD, we chose a variation of the box-counting method^17^. As a way of introducing FD, note that the number of tiles, each with area *L*^2^, needed to cover a floor is proportional to 1/*L*^2^, where the exponent “2” is due to the floor being two-dimensional. Now consider a 2D image covered with a grid of squares (each with area *L*^2^) as described above, with 1’s and 0’s corresponding to the answers to a binary question. The number *n*(*L*) of squares with ‘1’ will be proportional to (1/*L*^δ^) where the exponent δ is one type of FD and is ≤2^17^. If the system is self-similar and fractal over a range of length scales L, n(L) will follow a power law: *n*(*L*) = *A*/(*L*^δ^), and the exponent δ will remain constant as *L* varies. The constant of proportionality, *A*, depends on the size of the tissue that dictates the number of boxes covering the tissue. To avoid this, we define the FD as follows. In a plot of log [*n*(*L*)] versus log[*A*/*L*], where *A* is a constant, the FD is the slope *s* of a line fit through the points using linear regression with a least-squares fit, i.e., *s*(*L*) = −d[log *n*(*L*)]/d[log *L*]. (Note that unlike the more common definition of the box-counting FD, the limit *L*→0 is not taken because of our interest in the distribution of individual cells at different length scales.) The FD over a range of values of *L* is determined for each patient and then averaged over all patients with a given clinical outcome.

#### Occupancy and FDs of lymphocytes in cancer islands

In the calculations of the occupancy and FD with a grid of squares covering the tissue, squares with at least one CD20^+^ B cell (or T cell) in a cancer cell island were assigned a ‘1’, while squares covering stroma had zeros. The fraction of squares with 1’s, i.e., the occupancy, was the number of squares with 1’s divided by the total number of squares covering the image (including the stroma). This can easily be generalized to calculating the occupancy and FD of lymphocytes in the stroma or in the whole tissue.

#### Normalization of the distribution of cluster sizes

In Fig. 6e, f, the quantity on the *y*-axis is *P*_o,i_(*s*)/*N*_o,i_ where s is the number of lymphocytes in an LC, *N*_o,i_ is the total number of lymphocytes in LCs of type i (i = pure T LCs or heterotypic LCs) and outcome o (o = good or poor), and *P*_o,i_(*s*) is the number of LCs (of type i and outcome o) of size *s*. The normalization condition is

1 $$\frac{1}{{N_{o,i}}}\mathop {\sum }\limits_{s{\prime} = 5}^\infty P_{o,i}\left ({s{^\prime}} \right) \cdot s{^\prime} = 1$$

since the sum over *s’* of the fraction of lymphocytes in a cluster of size *s’* must equal unity. Blue line is for good outcome and red line is for poor outcome. Insets: Cumulative fraction of lymphocytes that belong to LCs of size s or smaller versus s. The x-axis is on a logarithmic scale. The cumulative fraction is given by the expression

2 $$f\left( s \right) = \frac{1}{{N_{o,i}}}\mathop {\sum }\limits_{s{\prime} = 5}^s P_{o,i}\left( {s{^\prime}} \right) \cdot s{^\prime}$$

### Plots

Throughout the paper, the threshold for RFS plots was systematically chosen so that two thirds of the patients are in the upper curve and one third are in the lower curve. These fractions correspond to the proportions of good and poor outcome patients, respectively.

### Reporting summary

Further information on research design is available in the Nature Research Reporting Summary linked to this article.

## Supplementary information

Reporting Summary

## Data Availability

The data generated and analysed during this study are described in the following data record: 10.6084/m9.figshare.14575734^47^. All data are contained in the R Data file ‘PatientPointPatterns.rds’, which is openly available with the data record. The file contains a list of 36 marked point patterns in ppp format, usable by the R package “spatstat”. Each pattern corresponds to a single patient. Relevant marks include “Tissue.Category” (marks points as belonging to cancer islands or stroma) and “Phenotype” (marks points as cancer, stromal, or immune cells). X and Y coordinates have units of μm.

## Code availability

All custom codes used in this study are available upon request.
