# Supplementary material for: Spatial distribution of B cells and lymphocyte clusters as a predictor of triple-negative breast cancer outcome
Source: NPJ Breast Cancer. 2021 Jul 1;7:84. doi: 10.1038/s41523-021-00291-z (PMC8249408; doi:10.1038/s41523-021-00291-z)
Supplement: Supplementary file 1 — Reporting Summary [file 41523_2021_291_MOESM1_ESM.pdf]

nature research

Corresponding author(s): Clare Yu

Last updated by author(s): Mar 24, 2021

Reporting Summary

Nature Research wishes to improve the reproducibility of the work that we publish. This form provides structure for consistency and transparency in reporting. For further information on Nature Research policies, see our Editorial Policies and the Editorial Policy Checklist.

Statistics

For all statistical analyses, confirm that the following items are present in the figure legend, table legend, main text, or Methods section.

n/a	Confirmed	
☐	☒	The exact sample size (n) for each experimental group/condition, given as a discrete number and unit of measurement
☐	☒	A statement on whether measurements were taken from distinct samples or whether the same sample was measured repeatedly
☐	☒	The statistical test(s) used AND whether they are one- or two-sided<br>*Only common tests should be described solely by name; describe more complex techniques in the Methods section.*
☒	☐	A description of all covariates tested
☒	☐	A description of any assumptions or corrections, such as tests of normality and adjustment for multiple comparisons
☐	☒	A full description of the statistical parameters including central tendency (e.g. means) or other basic estimates (e.g. regression coefficient) AND variation (e.g. standard deviation) or associated estimates of uncertainty (e.g. confidence intervals)
☐	☒	For null hypothesis testing, the test statistic (e.g. F, t, r) with confidence intervals, effect sizes, degrees of freedom and P value noted<br>*Give P values as exact values whenever suitable.*
☒	☐	For Bayesian analysis, information on the choice of priors and Markov chain Monte Carlo settings
☒	☐	For hierarchical and complex designs, identification of the appropriate level for tests and full reporting of outcomes
☐	☒	Estimates of effect sizes (e.g. Cohen's d, Pearson's r), indicating how they were calculated

Our web collection on statistics for biologists contains articles on many of the points above.

Software and code

Policy information about availability of computer code

Data collection	All the images were acquired using the Vectra 3.0 Automated Quantitative Pathology Imaging System (Akoya Biosciences) and commercially available software packages (inForm, Akoya Biosciences) were used to identify each cell, define its type (cancer or specific immune), and assign it Cartesian coordinates. Using an automated tissue segmenter algorithm built in inForm®, we further divided the images into areas of cancer islands and stroma based on anti-pan cytokeratin antibody staining.
Data analysis	We processed the data using in-house custom developed software (RStudio) and the R packages “spatstat” for point patterns, “dbscan” for cluster analysis, and “EBImage” for raster images.

For manuscripts utilizing custom algorithms or software that are central to the research but not yet described in published literature, software must be made available to editors and reviewers. We strongly encourage code deposition in a community repository (e.g. GitHub). See the Nature Research guidelines for submitting code & software for further information.

Data

Policy information about availability of data

All manuscripts must include a data availability statement. This statement should provide the following information, where applicable:

- Accession codes, unique identifiers, or web links for publicly available datasets
- A list of figures that have associated raw data
- A description of any restrictions on data availability

All data are available upon request.

Field-specific reporting

Please select the one below that is the best fit for your research. If you are not sure, read the appropriate sections before making your selection.

☒ Life sciences ☐ Behavioural & social sciences ☐ Ecological, evolutionary & environmental sciences

For a reference copy of the document with all sections, see nature.com/documents/nr-reporting-summary-flat.pdf

Life sciences study design

All studies must disclose on these points even when the disclosure is negative.

Sample size	This study involved archival samples that matched specific criteria.
Data exclusions	Patients not fitting the criteria for poor outcome (recurrence within 3 years of surgical resection) or good outcome (no recurrence for at least 5 years of surgical resection) were excluded.
Replication	Replication is not relevant because this was a retrospective study.
Randomization	Randomization is not relevant because this was a retrospective study.
Blinding	Blinding is not relevant because this was a retrospective study.

Reporting for specific materials, systems and methods

We require information from authors about some types of materials, experimental systems and methods used in many studies. Here, indicate whether each material, system or method listed is relevant to your study. If you are not sure if a list item applies to your research, read the appropriate section before selecting a response.

Materials & experimental systems

n/a	Involved in the study	
☐	☒	Antibodies
☒	☐	Eukaryotic cell lines
☒	☐	Palaeontology and archaeology
☒	☐	Animals and other organisms
☐	☒	Human research participants
☒	☐	Clinical data
☒	☐	Dual use research of concern

Methods

n/a	Involved in the study	
☒	☐	ChIP-seq
☒	☐	Flow cytometry
☒	☐	MRI-based neuroimaging

Antibodies

Antibodies used	Multi-color immunostaining was performed including anti-pan cytokeratin (AE1/AE3, Dako), anti-CD8 (SP16, Biocare), anti-CD3 (Polyclonal, Dako), anti-FOXP3 (236A/E7, Biocare) and anti-CD20 (L26, Dako).
Validation	Each antibody was individually validated and titrated against appropriate positive control samples.

Human research participants

Policy information about studies involving human research participants

Population characteristics	Given in Table 1 of manuscript.
Recruitment	No patients were specifically recruited for this retrospective study.
Ethics oversight	Specimens were identified through an IRB-approved protocol via the City of Hope (COH) Biospecimen Repository which is funded in part by the National Cancer Institute.

Note that full information on the approval of the study protocol must also be provided in the manuscript.
